# Bibliometric and visual analysis of radiomics for evaluating lymph node status in oncology

**DOI:** 10.3389/fmed.2024.1501652

**Published:** 2024-11-14

**Authors:** Gui-Wen Lyu, Tong Tong, Gen-Dong Yang, Jing Zhao, Zi-Fan Xu, Na Zheng, Zhi-Fang Zhang

**Affiliations:** ^1^Department of Radiology, The Third People's Hospital of Shenzhen, The Second Affiliated Hospital of Southern University of Science and Technology, Shenzhen, China; ^2^Department of Ultrasound, Shenzhen People’s Hospital, The Second Clinical Medical College, Jinan University, The First Affiliated Hospital, Southern University of Science and Technology, Shenzhen, China; ^3^Department of Pathology, Shenzhen University Medical School, Shenzhen, China

**Keywords:** radiomics, lymph node, oncology, artificial intelligence, bibliometric analysis

## Abstract

**Background:**

Radiomics, which involves the conversion of digital images into high-dimensional data, has been used in oncological studies since 2012. We analyzed the publications that had been conducted on this subject using bibliometric and visual methods to expound the hotpots and future trends regarding radiomics in evaluating lymph node status in oncology.

**Methods:**

Documents published between 2012 and 2023, updated to August 1, 2024, were searched using the Scopus database. VOSviewer, R Package, and Microsoft Excel were used for visualization.

**Results:**

A total of 898 original articles and reviews written in English and be related to radiomics for evaluating lymph node status in oncology, published between 2015 and 2023, were retrieved. A significant increase in the number of publications was observed, with an annual growth rate of 100.77%. The publications predominantly originated from three countries, with China leading in the number of publications and citations. Fudan University was the most contributing affiliation, followed by Sun Yat-sen University and Southern Medical University, all of which were from China. Tian J. from the Chinese Academy of Sciences contributed the most within 5885 authors. In addition, *Frontiers in Oncology* had the most publications and transcended other journals in recent 4 years. Moreover, the keywords co-occurrence suggested that the interplay of “radiomics” and “lymph node metastasis,” as well as “major clinical study” were the predominant topics, furthermore, the focused topics shifted from revealing the diagnosis of cancers to exploring the deep learning-based prediction of lymph node metastasis, suggesting the combination of artificial intelligence research would develop in the future.

**Conclusion:**

The present bibliometric and visual analysis described an approximately continuous trend of increasing publications related to radiomics in evaluating lymph node status in oncology and revealed that it could serve as an efficient tool for personalized diagnosis and treatment guidance in clinical patients, and combined artificial intelligence should be further considered in the future.

## 1 Introduction

Lymphatic metastasis is a common pathway for the spread of malignant tumors, and has significant implications for prognosis and treatment selection. The lymph node status is a crucial indicator for assessing the prognosis and treatment outcomes of clinical cancer patients (1–4). Traditional imaging assessment methods have limitations in evaluating tumor lymphatic metastasis and lymph node status (5), fortunately, in recent decade, radiomics, combined with machine learning and artificial intelligence (AI) algorithms, has shown great potential in this field (6–8). Through radiomics, a more accurate assessment of the risk of tumor lymphatic metastasis, prediction of the status of lymph node metastasis, and provision of a more reliable basis for individualized treatment planning are achievable (9, 10).

Radiomics is a burgeoning field in medical imaging that focuses on extracting and analyzing numerous quantitative features from radiological images. These features encompass a wide range of data, including shape, intensity, texture, and wavelet features, among others, providing valuable insights into the underlying pathophysiology of diseases (6, 11). By leveraging advanced computational techniques and machine learning algorithms, radiomics aims to transform standard medical images into high-dimensional data for improved disease characterization, prognosis, and treatment response assessment (12–14). The application of radiomics has shown promising potential in various types of cancer, such as colorectal cancer, breast cancer, lung cancer, papillary thyroid cancer, *et cetera*, offering a non-invasive and personalized approach to medical diagnosis and treatment evaluation (8, 9, 15, 16).

Bibliometric analysis has become an essential tool for research evaluation, knowledge mapping, and trend analysis in various academic disciplines. By quantitatively analyzing publication data, citation patterns, and collaboration networks, bibliometric analysis provides valuable insights into the research landscape (17). In recent years, the visualization of bibliometric data has gained prominence, with tools including VOSviewer, or R software, enabling researchers to create interactive and informative visualizations (18–20). VOSviewer, a popular software tool for constructing and visualizing bibliometric networks, allows users to explore keyword co-occurrence, and collaboration networks in a user-friendly interface (21). Meanwhile, R software package, with its diverse range of packages and libraries, offers flexibility in customizing visualizations and conducting advanced bibliometric analysis (22).

This bibliometric analysis aimed to delve into the global application of radiomics in evaluating the lymph node state of tumors, showcasing its capabilities in uncovering research trends, identifying key players in the field, and mapping knowledge domains. By exploring the visualization features of VOSviewer and R software packages, researchers can gain deeper insights into the scholarly landscape and make informed decisions based on data-driven analysis.

## 2 Methods

### 2.1 Data sources and search strategies

**Figure 1:**
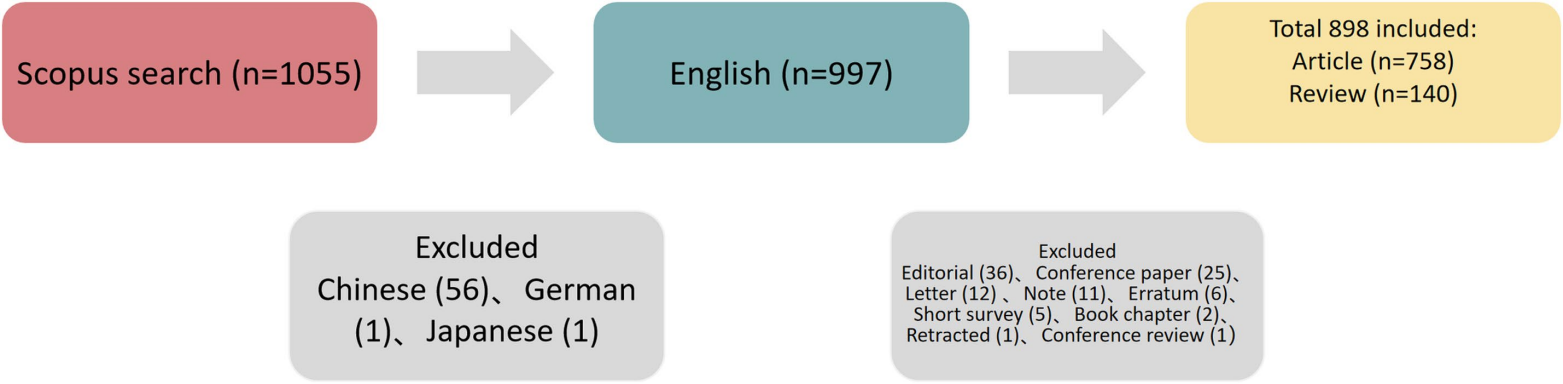
Study flow diagram.

The present study collected data from the Scopus database, with the search period setting between 2012 and 2023, updated on August 1, 2024. The search terms were as follows: TITLE-ABS-KEY = (“radiomic” OR “radiomics”) AND TITLE-ABS-KEY = (“lymph” AND “node”) AND TITLE-ABS-KEY = (“tumor” OR “cancer” OR “carcinoma”OR “oncology”) AND PUBYEAR > 2012 AND PUBYEAR < 2024. Among the diverse types of documents (articles, book chapters, conference papers, conference reviews, editorials, letters, notes, reviews, and short surveys), only articles and reviews written in English were included. In total, 758 articles and 140 reviews were retrieved for further analysis. The strategy for enrolling and retrieving the documents was shown in Figure 1.

### 2.2 Data acquisition and bibliometric analysis

Text data were downloaded, and bibliometric indicators, such as the H-index, were utilized to evaluate the scholarly achievements of individuals, along with the impact factors of relevant academic journals. Bibliometric and visual analyses were conducted using VOSviewer and the R package. Microsoft Office Excel 2019 was used as a supplement to create the statistical charts. To better show the co-occurence, the keywords were combined, including “lymph nodes AND lymph node,” “neoplasm staging AND cancer staging,” “breast tumor AND breast cancer,” “breast neoplasms AND breast cancer,” “nomograms AND nomogram,” “fluorodeoxyglucose f 18 AND 18F-FDG-PET,” “lung neoplasms AND lung tumor,” “tomography, x-ray computed AND x-ray computed tomography,” “humans AND human,” “nuclear magnetic resonance imaging AND magnetic resonance imaging,” “retrospective studies AND retrospective study,” and “positron emission tomography-computed tomography AND positron emission tomography computed tomography,” respectively.

## 3 Results

### 3.1 Main information

Based on the study flow diagram illustrated in Figures 1, 898 documents published between 2015 and 2023 were retrieved, containing 19,756 citations with an average of 22 citations per paper. The annual growth rate was 100.77% and the average age of the documents was 2.55. In total, 248 journals, 61 countries, 3657 affiliations, and 5,885 authors contributed 4,317 keywords plus were included.

### 3.2 Annual trends in the number of publications

**Figure 2:**
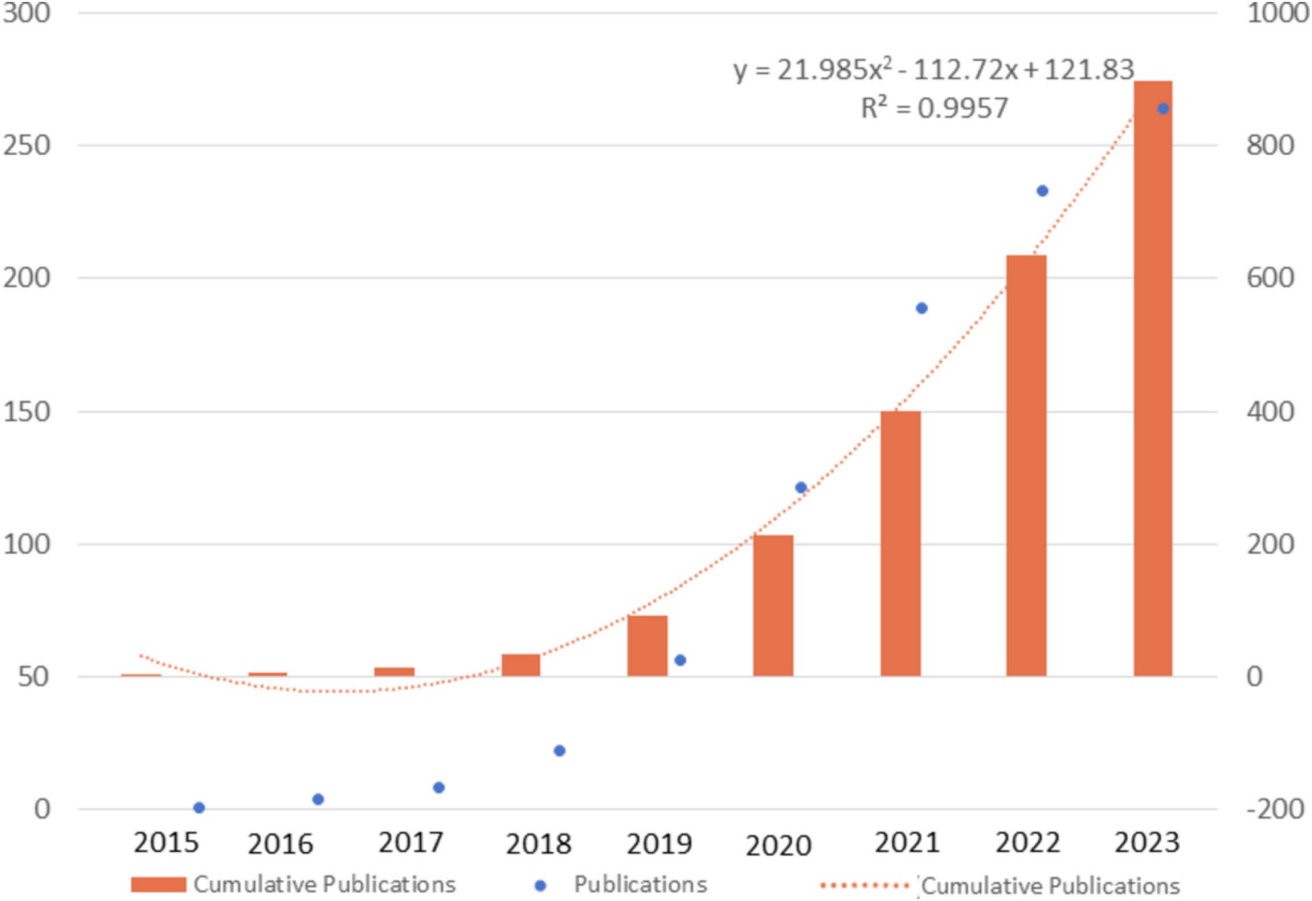
Trends of publications between 2015 and 2023.

In general, there were 99.8 annual publications between 2015 and 2023. As depicted in Figure 2, the number of publications in this field continued to sostenuto, especially with a dramatic increase from 2020, manifested as the number of publications (NP) exceeding 100 and the cumulative number of publications doubling every 1 or 2 years. The curve of the cumulative number of publications fitted the quadratic function curve with a goodness-of-fit *R*^2^ of 0.9957, indicating that the total number of publications will grow steadily in the coming years.

### 3.3 Countries and areas analysis

**Figure 3:**
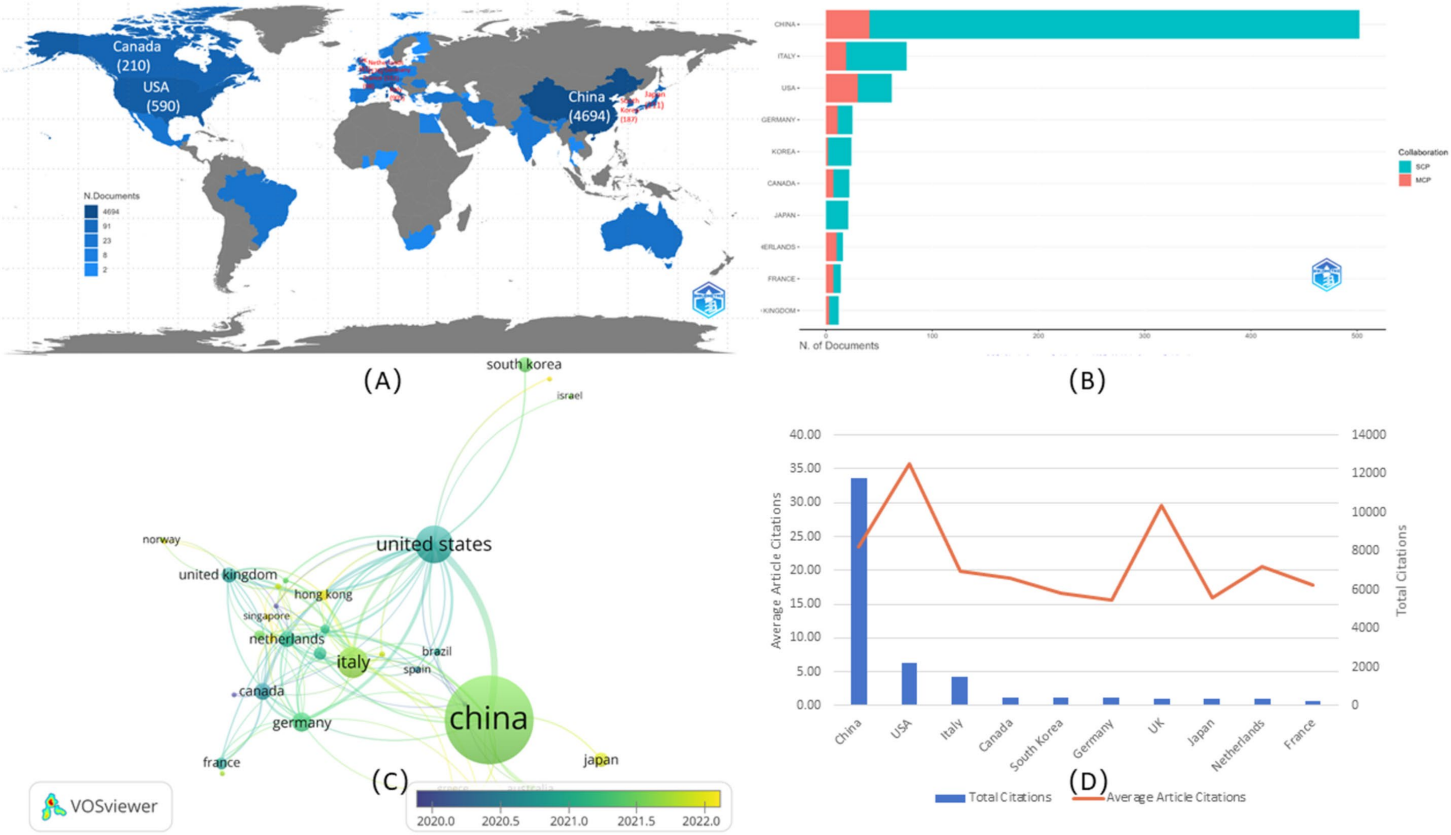
Countries analysis. **(A)** Global geographic map. The color shading indicated the amount of publications, with the label of top 10 counties. **(B)** International collaboration of top 10 countries. The yellow color represented the single country publication (SCP), and the blue color represented the multiple country publication (MCP). **(C)** Overlay map of co-authorship of the dominating countries. The size represented the amount of publications, and the yellow color represented documents published mainly from 2022. **(D)** The citations of top 10 countries. The blue columns represented the total citations, and the red curve represented the average article citations.

The analysis of publications, citations, and collaboration for various countries and areas indicated the degree of importance in the research field of the countries as well as the degree of effect. As depicted in Figure 3A, the top 10 contributing countries were mainly from Asia, such as China (502, 4694), South Korea (24, 187), and Japan (21, 211); North America, such as the USA (62, 590) and Canada (22, 210); and European countries, including Italy (76, 905), Germany (25, 312), the Netherlands (16, 176), France (14, 98), and the UK (12, 81). The former referred to documents based on the corresponding author, while the latter referred to documents based on every co-author. The disparity in the numbers within one country was mainly due to international collaboration among authors, as the data showed that the co-authors per document was 8.92, taken account to totally 6 publications with single-author. Among these countries, China had the highest contribution based on the number of publications, followed by the USA and Italy. For the international collaboration depicted in Figure 3B, within the top 10 contributing countries, the most single-country publications (SCP) were from China (461, 51.33%), followed by Italy (57, 6.35%), and the USA (32, 3.56%), while the most multiple-country publications (MCP) were from China (41, 4.57%), followed by the USA (30, 3.34%), and Italy (19, 2.12%). Moreover, for the ratio of MCP and NP, the prominent country among the top 10 countries was Netherlands with the number of 62.5%, indicating higher international collaboration, while, the prominent countries among all the included countries were Brazil, and India, with the number of 66.7%, mainly due to only 3 documents published for each country. The detailed parameters of the top 24 countries, including the number of publications, SCP, MCP, Frequency (%), and MCP Ratio were listed in Supplementary Table 1. The VOSviewer overlay map in Figure 3C showed that 36 countries and areas had cooperated with others to publish at least two documents. Among them, China cooperated with 13 countries and areas, including the USA, Japan, Australia, the Russian Federation, the UK, Italy, the Netherlands, Switzerland, Canada, Germany, South Africa, Hong Kong, and Taiwan; the most significant link was between China and the USA. The second-largest contributing country, representing a larger glaucous node, was the USA. Compared to China, the USA had more co-authorship countries. In total, 24 countries and areas were involved, including South Korea, Egypt, Israel, Romania, Denmark, Singapore, Austria, Mexico, Brazil, Spain, Thailand, Slovenia, and France, which had no collaboration with China. Similarly, China was the most crucial cooperative country in the USA. The yellow nodes, represented by Norway, Hong Kong, Singapore, the Russian Federation, and Greece, were emerging as novel contributing countries. As another crucial indicator, the cited times depicted in Figure 3D showed that China had the highest total citations, followed by the USA and Italy, mainly because of their higher number of publications. Therefore, the average number of article citations showed a better evaluation of the scientific influence of each country. Among the countries involved, the USA (35.8) had the highest average article citations, followed by the UK (29.6) and China (23.5). Apart from these 10 countries, Norway (30.0), Switzerland (27.4), and Australia (25.3) had higher average article citations. The detailed parameters of the top 25 countries, including the total citations and average article citations, were listed in Supplementary Table 2.

### 3.4 Affiliations and authors analysis

**Figure 4:**
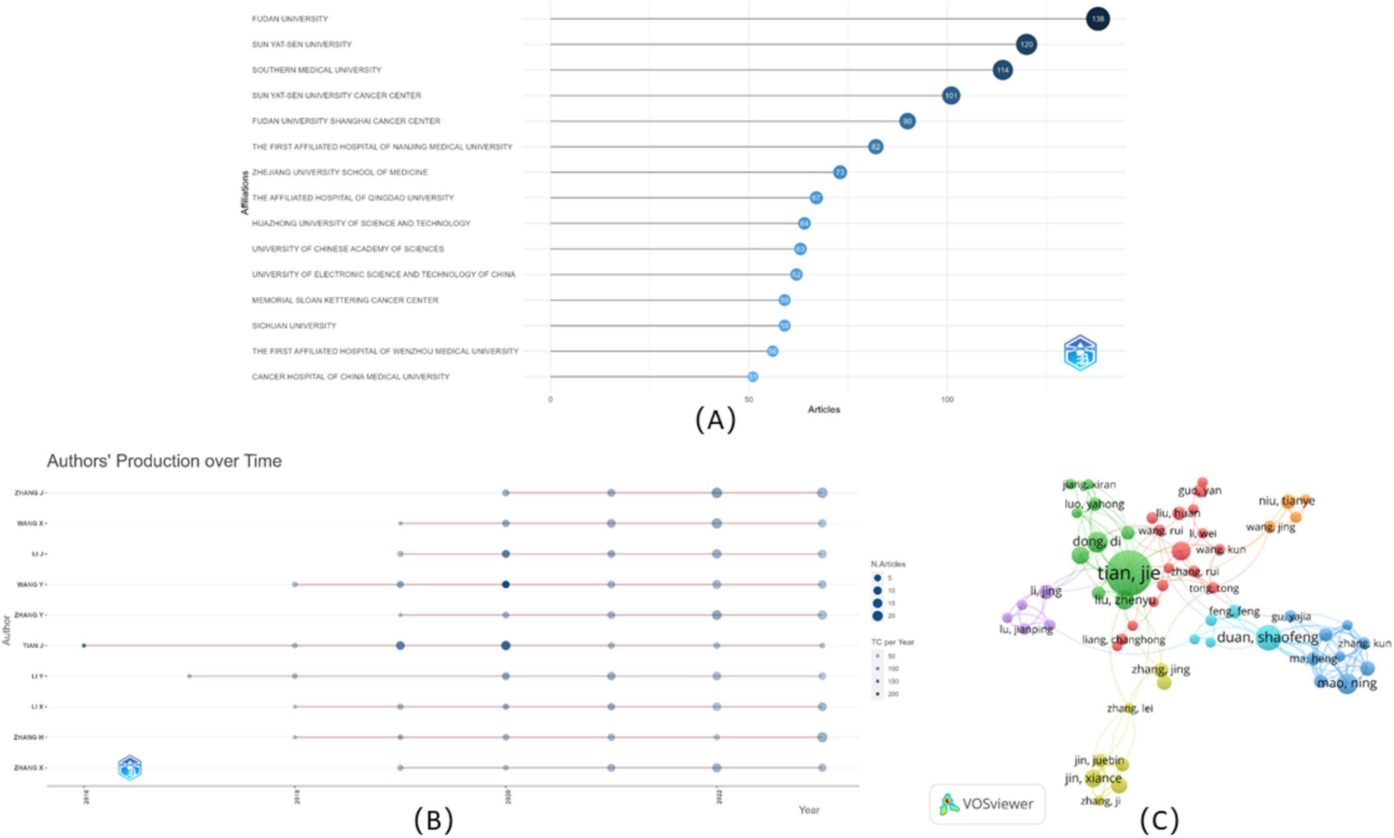
Visualization of affiliations and authors. **(A)** Top 15 affiliations analysis. **(B)** Top 10 authors’ publications over time. **(C)** Collaboration analysis among authors.

The top 15 affiliations with the most publications were shown in Figure 4A. Almost all affiliations were from China, except for the Memorial Sloan Kettering Cancer Center in the USA, which was ranked 12th. Additionally, all had published at least 50 documents, and four affiliations had published more than 100 documents. Among the top three affiliations, Fudan University ranked 1st published its first document in 2015 (23), with an annual publication reaching 15, while it published 45 in 2020 and 43 in 2023. Sun Yat-Sen University ranked 2rd published its first document in 2017 (24), with an annual publication of 17, while it published 39 in 2021. Southern Medical University ranked 3th in its first document in 2016 (9), with an annual publication of 14.

The 10 topmost authors with the highest number of publications were illustrated in Figure 4B, along with their publications over time. Zhang J. ranked 1st participated in 54 publications, who had published since 2020 (25), followed by Wang X. with 47 publications. Among the top 10 authors, Tian J. published the first document in 2016 (9), which had the highest number of citations to date. As shown in Table 1, Tian J. made a prominent contribution due to his highest total citations (3330), the highest average citations per article (87.63), and the highest average citations per year (416.25). In addition to Wang X., Li J., Zhang Y., and Zhang X. published their first documents in 2019 (26–28), Wang Y. and Li X. published their first documents in 2018 (29, 30). Moreover, some of them had relative collaboration to publish, such as Wang X. and Zhang Y. published their first documents in 2019 (26). Additionally, the co-authorship network was also explored in Figure 4C. Among the 5885 authors, 90 met the thresholds, setting the minimum number of documents of an author as five, and the largest set of connected items consisted of 58 items, instead of all involved items, because some of the 90 items were not connected to each other. Collaboration among authors showed weak relationships among collaborators, suggesting the need for greater collaboration in the future.

**Table 1:** Top 10 authors with high impact.

Authors	H-index	TC	NP	ACPA	PY_start	ACPY
Tian J	25	3330	38	87.63	2016	416.25
Li J	17	1128	43	26.23	2019	225.60
Wang Y	17	1861	43	43.28	2018	310.17
Zhang J	16	681	54	12.61	2020	170.25
Zhang L	16	891	31	28.74	2018	148.50
Li H	15	862	24	35.92	2015	95.78
Wang X	15	749	47	15.94	2019	149.80
Li Y	14	1111	36	30.86	2017	158.71
Liu Z	14	1003	23	43.61	2018	167.17
Zhang X	14	563	34	16.56	2019	112.60

### 3.5 Journals and keywords analysis

The R software package was used to analyze the journals published in these documents to explore and identify the most prominent and productive journals. These documents were published in 248 well-reputed academic journals. The 10 highest-ranking journals in terms of the NP were listed in Table 2. Among them, five journals were categorized as radiology, nuclear medicine, and medical imaging; four were categorized as oncology; and the remaining one was categorized as medicine, general, and internal. Among these, six were ranked Q1, three were ranked Q2, and one was ranked Q3. The open-access journal *Frontiers in Oncology* occupied the top position with 160 publications, followed by and *Cancers* and *European Radiology* with 51 and 41 publications, respectively. An analysis of the publications in each journal over time showed that four journals initially published in 2018, such as *Frontiers in Oncology*, *European Radiology*, *Journal of Magnetic Resonance Imaging*, and *European Journal of Radiology*, meanwhile, *Abdominal Radiology* published their first documents in 2021. Additionally, a dramatic increase in the number of publications on *Frontiers in Oncology* had been observed, especially in 2021 and 2022.

**Table 2:** Top 10 most contributing journals.

Journals	IF	Category	2018	2019	2020	2021	2022	2023
Frontiers in Oncology	3.5	Q2 (107/322)	1	7	27	77	134	160
Cancers	4.5	Q1 (78/322)	0	0	6	21	34	51
European Radiology	4.7	Q1 (22/204)	1	7	13	25	32	41
Journal of Magnetic Resonance Imaging	3.3	Q1 (42/204)	1	9	13	14	17	22
Academic Radiology	3.8	Q1 (38/204)	0	0	3	5	11	19
British Journal of Radiology	1.8	Q3 (113/204)	0	1	4	13	15	19
European Journal of Radiology	3.2	Q1 (46/204)	1	5	8	12	16	18
Diagnostics	3.0	Q1 (58/325)	0	0	1	4	10	17
Abdominal Radiology	2.3	Q2 (85/322)	0	0	0	4	10	16
Cancer Imaging	3.5	Q2 (107/322)	0	2	8	9	12	14

**Figure 5:**
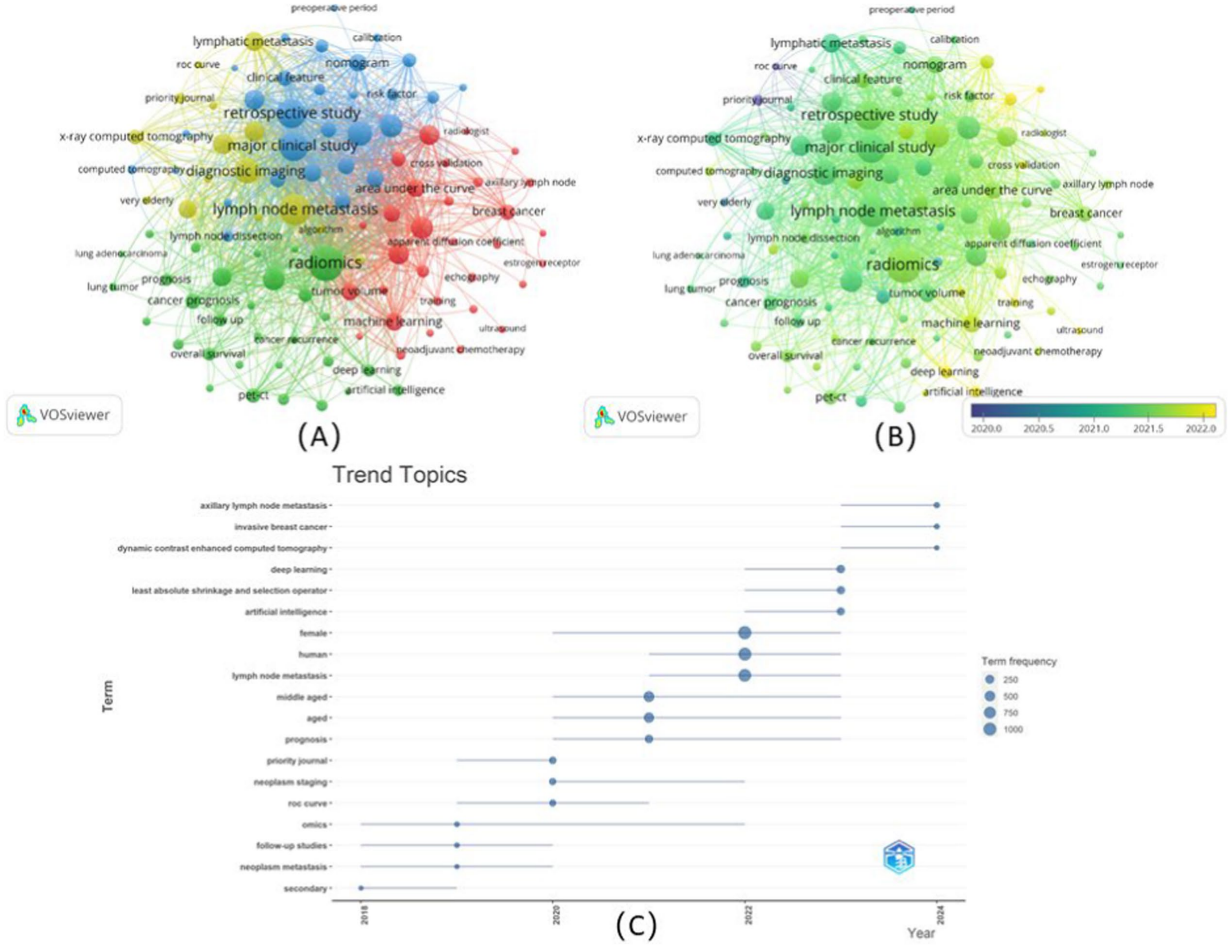
Co-occurrence network analysis of keywords. **(A)** The keywords were divided into four categories according to different colors: category 1(red), category 2(green), category 3(blue), and category 4(yellow), with the size of the nodes indicated the frequency of occurrence; **(B)** Visualization of the keyword co-occurrence overlay according to the average years of publication. Keywords colored yellow represented appearing later than those dark blue; **(C)** Top 20 keywords for burst detection each year.

Totally 65 keywords of these publications (including Author Keywords and Index Keywords) were analyzed and visualized based on keyword co-occurrence and burst detection (Figure 5). The results showed that four clusters were divided based on the strength of their relationship (Figure 5A). In brief, Cluster 1 (red) mainly concentrated on the study of the diagnosis combined radiomics and pathology focusing on breast cancer, with the prevalent keyword being “histopathology,” “breast cancer,” and “diagnostic test accuracy,” Cluster 2 (green) focused on the prognosis based on CT/PET-CT mainly for lung cancer assessment, with the prevalent keyword being “cancer prognosis,” “cancer survival,” and “lung tumor,” Cluster 3 (blue) focused on the predictive value especially on uterine cervix cancer and CA19-9 elevating related cancers, with the prevalent keyword being “prediction,” “retrospective study,” and “CA 19–9 antigen,” while Cluster 4 (yellow) mainly focused on the comparative studies of lymphatic metastasis on aged patients. The keywords were color-coded into different types according to the average year of publication using VOSviewer (Figure 5B). Research areas, such as radiomics, lymph node metastasis, major clinical studies, and retrospective studies illustrated around 2021 were the primary topics. Moreover, the keywords related to the AI, such as “deep learning” and “machine learning,” rapidly rose compared to inchoate “ROC curve,” and suggested the increasingly concerted efforts in pushing artificial intelligence technology to clinical use and to impact future directions in cancer care. Twenty keywords, burst intensity, burst duration, and burst time, were also assessed (Figure 5C). In the original stage, “neoplasm metastasis,” “neoplasm staging,” and “ROC curve” were the focus of research; however, in recent years, “artificial intelligence,” and “dynamic contrast-enhanced computed tomography” become emerging keywords, suggesting the clinical use of novel technology and methods.

### 3.6 Global citation score analysis

Based on the analysis of the database, the top 10 cited documents, with 3,445 citations, up to 17.44% of the total citations, were depicted in Table 3. Among them, only two articles ranked 2rd and 9th were published by authors from the USA (31, 32), and the others were all published by authors from China. The article “Development and validation of a radiomics nomogram for preoperative prediction of lymph node metastasis in colorectal cancer” published in Journal of Clinical Oncology in 2016 (9), ranked 1st with 1333 citations. For the article ranked 2nd, “Predicting response to cancer immunotherapy using non-invasive radiomic biomarkers” published in Annals of Oncology in 2019 with 366 citations (31). Moreover, the article “Deep learning radiomics can predict axillary lymph node status in early-stage breast cancer” ranked 3rd, published in Nature of Communications in 2020 with 343 citations (15). The article “A radiomics nomogram for the preoperative prediction of lymph node metastasis in bladder cancer”ranked 4th also had citation over 300 times, which was published in Clinical Cancer Research in 2017 (24). No hard to see, these articles were related to various types of cancer, including colorectal cancer, lung cancer, breast cancer, and bladder cancer, respectively. Additionally, apart from the breast cancer to be mentioned time after time (15, 33, 34), among the top 10 cited article, others were associated with other types of cancer be apt to lymphatic metastasis, such as gastric cancer (35), biliary tract cancer (26), and papillary thyroid carcinoma (36). For the radiomics nomograms, deep learning was the popular nomogram (15, 34, 36), and the transfer learning (36), which also been used in 2020, suggested that the development of the algorithms to achieve the goal that offering a repertoire of “expert” experiences in disease interpretation.

**Table 3:** Top 10 cited publications with total and annual citations.

Rank	Title	Pub. year	2016	2017	2018	2019	2020	2021	2022	2023	2024	Sub total
1	Development and validation of a radiomics nomogram for preoperative prediction of lymph node metastasis in colorectal cancer	2016	2	34	87	188	219	249	244	196	114	1333
2	Predicting response to cancer immunotherapy using noninvasive radiomic biomarkers	2019	0	0	0	10	58	97	76	83	42	366
3	Deep learning radiomics can predict axillary lymph node status in early-stage breast cancer	2020	0	0	0	0	6	56	87	105	89	343
4	A radiomics nomogram for the preoperative prediction of lymph node metastasis in bladder cancer	2017	0	0	7	51	74	62	45	43	26	308
5	Deep learning radiomic nomogram can predict the number of lymph node metastasis in locally advanced gastric cancer: an international multicenter study	2020	0	0	0	0	9	53	50	75	48	235
6	Preoperative prediction of sentinel lymph node metastasis in breast cancer based on radiomics of T2-weighted fat-suppression and diffusion-weighted MRI	2018	0	0	6	32	38	37	44	20	18	195
7	Deep learning vs. radiomics for predicting axillary lymph node metastasis of breast cancer using ultrasound images: do not forget the Peritumoral region	2020	0	0	0	0	4	33	63	48	27	175
8	Biliary tract cancer at CT: a radiomics-based model to predict lymph node metastasis and survival outcomes	2019	0	0	0	11	29	40	37	27	25	169
9	Radiomic-based pathological response prediction from primary tumors and lymph nodes in NSCLC	2017	0	6	13	19	20	36	32	24	14	164
10	Lymph node metastasis prediction of papillary thyroid carcinoma based on transfer learning radiomics	2020	0	0	0	0	0	25	39	49	44	157
		Total citation	2	40	113	311	457	688	717	670	447	3445

The top 10 cited publications were analyzed based on their global citation scores per year. Nine of the top 10 publications were articles related to the relatively high impact and citation count of high-level journals. Higher citations range from 2021 to 2023, with 670 to 717 citations. However, for some articles published earlier, the most cited articles were from 2019 to 2022, such as “Preoperative prediction of sentinel lymph node metastasis in breast cancer based on radiomics of T2-weighted fat-suppression and diffusion-weighted MRI” published in 2018 (33). Especially, the article “Radiomic-Based Pathological Response Prediction from Primary Tumors and Lymph Nodes in NSCLC” published in 2017 (32), showed the same tendency of being cited as the 1st ranked article published in 2016.

### 3.7 Subject areas and funding sponsors analysis

As an interdisciplinary field, the documents referred to multiple subject areas, mainly medicine (824, 91.76%) and biochemistry, genetics, and molecular biology (374, 41.65%), which were ranked 1st and 2rd, respectively. The top 10 subject areas were health professionals (60, 6.68%), computer science (23, 2.56%), physics and astronomy (23, 2.56%), engineering (22, 2.45%), multidisciplinary (17, 1.89%), immunology and microbiology (14, 1.56%), pharmacology, toxicology, and pharmaceutics (12, 1.34%), and dentistry (6, 0.67%). Besides these subject areas, all of them were shown in Supplementary Table 3.

A total of 159 global funding sponsors were involved, with the National Natural Science Foundation of China (217, 24.16%) ranked 1^st^, followed by the Ministry of Science and Technology of the People’s Republic of China (43, 4.79%), and the National Institutes of Health (43, 4.79%). Apart from these, among the top 10 funding sponsors based on the amount, two sponsors were from the USA, including the National Cancer Institute (37, 4.12%) and the U.S. Department of Health and Human Services (19, 2.12%), while the other five were all from China, with detailed information on funding sponsors with numbers over 10, as shown in Supplementary Table 4.

## 4 Discussion

The current status and trends in the development of radiomics-related research in lymph node state evaluation in oncology were analyzed for the first time using a bibliometric analysis. According to the Scopus database, the documents had been published since 2015 (23), which was 1 year earlier than the documents included in the Web of Science Core Collection database.

Since 2012, the concept of radiomics had been proposed to address this problem because of the huge potential for quantitative analysis of medical imaging to capture intratumoral heterogeneity in a non-invasive manner (6). With the development of automated and reproducible analysis methodologies, the annual growth rate of relevant original articles published from 2013 to 2018 was 177.82% (37). Another research showed that the annual production had increased steadily since 2016, and the estimated number of publications on radiomics would reach 2700 by the end of 2023 (38). As a branch of radiomics in oncology, our analysis found that the later beginning, but the similarly dramatic increase in the annual growth rate of the research on the evaluation of lymph node status.

Although the leading position of the USA in the global research of radiomics had been maintained for several years (37), the remarkable increase in scientific research output from China also positioned the country at the forefront of various fields, such as computer science, technology, and AI (39, 40), leading the way with a growing number of publications in the field of radiomics applied to evaluate lymph node status in oncology. This trend underscored China’s expanding influence and substantial contribution to global scientific knowledge. China’s commitment to research and development, along with significant investments in scientific infrastructure and talent, had propelled the nation to the forefront of scientific innovation, especially representing the New Generation Artificial Intelligence Development Plan of China (2015–2030) (40). The collaborative efforts between academia, industry, and government initiatives had created an environment conducive to groundbreaking discoveries and advancements, for special performance, sufficient funding supporting the research in this field, including multitudinous national and provincial funding moving beyond mentioned in our analysis, such as the National Natural Science Foundation of China, Ministry of Science and Technology of the People’s Republic of China, and so on, as shown in Supplementary Table 4. Consequently, China’s scientific achievements had shaped the global scientific landscape and made significant contributions to various fields, marking a transformative era in the country’s scientific endeavors.

Moreover, documents published in China also had the highest number of total citations, indicating a relatively high quality of publications in this research area and a good reference value. In view of another indicator, the average citation per article in the USA was better than one tally among the top 10 countries with higher publications. The visual analysis of the collaboration between countries showed that the collaboration between China and the USA was strong, whereas the links among other countries were much weaker. To better portray research and assess problems in this field, affiliations and scholars from all these countries drew on others’ merits to offset their own weaknesses and actively communicate and cooperate. Owing to China’s prominent contribution to the number of publications, most of the remarkable affiliations and authors were from China. It was worth mentioning, based on Figure 4, the Memorial Sloan Kettering Cancer Center in the USA also had more publications, including the first article published in 2015 (23), indicating that China plus the USA had a greater influence in this field. To identify the most prolific authors, the top 10 authors with the most publications were analyzed, as shown in Table 1. Most of the authors ranked high in the field of radiomics-related research on evaluating lymph node status published their first article during the first half of the decade, except Zhang J, who published his first article in 2020 and participated in the majority of publications, which was also depicted in Figure 4B. The visual network of the authors shown in Figure 4C suggested active collaboration between Tian J and other authors, which might had a higher impact, manifested as a higher H-index, ACPA, and ACPY. It was suggested that more effective academic exchanges between scholars accelerate progress, moving beyond the scholar himself but also research in the field.

The sources contributing to the publications in this field were counted using the R package, and 248 journals were assessed with the publications in *Frontiers in Oncology* (IF: 3.5, JCR:Q2) ranking 1st, which belonged to Frontiers Media. Among the top 10 journals, none had an impact factor of over five, mainly due to fewer citations, and focus was gained in journals in the categories of radiology, nuclear medicine, and medical imaging. In addition, open-access journals showed more advantages in the expanding outreach of this research field, such as *Frontiers in Oncology* and *Cancers*, but they also indicated that higher quality and better reference value of the journals should be involved in the future.

Unlike other studies, the studies involved in our analysis were mainly related to clinical patients, not animal or *in vivo* research, as shown in Figure 5A, with the keywords of “major clinical study,” and of “retrospective study.” Therefore, the “human” became the top occurrence keyword, even though it was not depicted in the figure, to reduce the heterogeneity of the occurrence numbers among the keywords. This research attempted to develop and verify the effectiveness of nomograms in clinical trials, which was a method of analyzing tumor characteristics by extracting quantitative features from medical images and had shown potential in the study of lymphatic metastasis across various tumor types, including lung, colorectal, head and neck, and pancreatic cancers. Moreover, the applicability of different types of imageological examinations for various types of cancers was discrepant, such as the prediction of lymph node metastasis by ultrasound and DWI in breast cancer (33, 34), and CT in colorectal cancer and gastric cancer (9, 27).

As shown in cluster 1, radiomics had made significant strides in advancing the understanding of lymphatic metastasis in breast cancer (33, 34). As breast cancer was prone to lymph node metastasis, the development of radiomics in the diagnosis and outcome prediction of breast cancer had been remarkable. To identify radiomic features for predicting lymph node metastasis in breast cancer, Liu et al. (41) successfully identified specific radiomic features that were highly predictive of lymph node metastasis in patients with breast cancer, allowing for more accurate diagnosis and treatment planning. They found that the DCE-MRI-based radiomics signature in combination with MRI ALN status was effective in predicting the LVI status of patients with invasive breast cancer before surgery (41). For risk stratification, novel radiomics-based models had been developed to stratify breast cancer patients based on their risk of lymph node metastasis, enabling personalized treatment strategies and improving patient outcomes (42). For the validation of radiomics in large-scale clinical studies, a multicenter clinical study by Yu et al. (43) validated the efficacy of radiomics in predicting lymph node metastasis in breast cancer, demonstrating its potential as a reliable and non-invasive diagnostic tool. Moreover, they found that significant changes in key radiomic features after neoadjuvant chemotherapy might be explained by changes in the tumor microenvironment, and the association between MRI radiomic features and tumor microenvironment features may reveal the potential biological underpinning of MRI radiomics (43). For integration of radiomics with molecular biomarkers, it had shown to enhance the predictive power of lymph node metastasis in breast cancer, providing a comprehensive approach to patient management. Pinker et al. (44) reviewed numerous studies about the radiogenomics in breast cancer, which might provide voxel-by-voxel genetic information for a complete, heterogeneous tumor or, in the setting of metastatic disease, set of tumors and thereby guide tailored therapy (44). As shown in cluster 2, in lung cancer, included lung adenocarcinoma, and non-small cell lung cancer, studies had demonstrated that imaging features could predict lymph node metastasis, with deep learning models improving prediction accuracy from CT images (45, 46). Zheng et al. (47) performed a systematic review, and found that the pooled AUROC of six studies that determined whether patients had lymph node metastases was 0.74, which suggested the models based on deep learning or radiomics had the potential to improve diagnostic accuracy for lung cancer staging (47). As shown in cluster 3, in CA-199 elevation related uterine cervix cancer, the results demonstrated MRI based radiomics signature could be used as a prognostic biomarker or non-invasive biomarker for preoperative assessment of lymph node status and potentially influence the therapeutic decision-making in early-stage cervical cancer patients (48, 49). The presence of pelvic and para-aortic lymph node metastases had a prognostic significance, and its detection was paramount to define the best treatment option. By extracting texture features from a polygonal ROI drawn on the primary lesion at baseline pelvic MR, they found that higher skewness or kurtosis in the main tumor was associated with lymph nodes involvement (50). As shown in cluster 4, there was no specific type of cancer that was mentioned. However, it emphasized the combination of radiomics and pathology. Yan et al. (51) constructed a comprehensive model based on clinicopathology, ultrasound, PET/CT, and PET radiomics, which could effectively improve the diagnostic efficacy of axillary lymph node metastasis in breast cancer (51).

Moving beyond the high frequency of co-occurrence keywords, other types of cancers had also been studied worldwide. For colorectal cancer, radiomics features had been utilized to assess the risk of lymphatic spread, effectively differentiating patients with and without metastasis through MRI analysis (52, 53). For head and neck cancer, the extraction of imaging features had been closely associated with lymphatic metastasis incidence and prognosis, with PET/CT images helping to identify high-risk patients (54, 55). In pancreatic cancer, radiomics had been used to evaluate the likelihood of lymphatic involvement, with CT image analysis providing insights into metastasis risk (56, 57). Up to date, by extracting and analyzing quantitative features from medical imaging, radiomics had improved risk stratification, enabled personalized medicine, and enhanced tumor characterization. These advances had provided valuable insights into lymphatic involvement and disease progression. While current research results are promising, further large-scale clinical trials are needed to validate the effectiveness and address challenges, such as data standardization and model interpretability, emphasizing the importance of multi-center collaborations and data sharing for advancing the integration of radiomics into clinical practice. However, challenges related to the data quality and interpretability of complex artificial intelligence-driven models persisted, highlighting the need for further development in these areas to fully leverage the potential of radiomics in clinical practice.

Undoubtedly, radiomics had shown significant progress in the clinical assessment and management of lymphatic metastases using ultrasound, CT, PET-CT, and MR. Although ultrasound imaging had been widely used for tumor evaluation, its application in radiomics for lymphatic metastasis in clinical oncology remains an emerging area of research. However, preliminary studies had demonstrated the potential of ultrasound-based radiomics for lymph node assessment in various cancers, including breast and thyroid cancer. These studies had indicated that certain ultrasound-derived radiomic features might have a predictive value in identifying lymphatic involvement. Radiomics models were valuable in predicting axillary lymph node metastasis in breast cancer, and for both CNNs and radiomics models, combining intratumoral, and peritumoral regions achieved significantly better performance (34). Cao et al. (58) presented a comprehensive overview that leveraging artificial intelligence with a focus on traditional machine learning (ML) algorithms and DL algorithms in thyroid cancer could help radiologists achieve more accurate and efficient imaging diagnosis and reduce their workload (58). As the field continues to evolve, further research and validation are needed to fully establish the role of ultrasound-based radiomics in the clinical assessment and management of lymphatic metastasis in cancer patients. Continued exploration in this area could potentially expand the scope of radiomics applications and contribute to a more comprehensive and multimodal approach to cancer evaluation and treatment. CT-based radiomics analysis had enabled the identification of imaging characteristics associated with lymph node metastasis in lung cancer, with machine learning algorithms improving the accuracy of convolutional neural network predicting metastatic spread (16, 45, 46). Additionally, CT imaging had been effectively utilized to assess the risk of lymphatic involvement in pancreatic cancer through the extraction of radiomics features (56, 57). Similarly, PET-CT imaging had been instrumental in identifying high-risk patients for lymph node metastasis in head and neck cancer, showcasing the potential of radiomics in refining treatment strategies (54). Radiomics involved extracting quantitative imaging features from MRI scans, which could provide valuable insights into tumor characteristics and behavior. For instance, studies had demonstrated that specific radiomic features derived from MRI could effectively differentiate between patients with and without lymph node metastasis in colorectal cancer and aid in accurate staging and treatment planning (9, 25, 52). Additionally, radiomics had been applied to assess lymphatic spread in other tumor types, such as breast and prostate cancers, and MRI had been used to identify subtle imaging biomarkers associated with metastasis (33, 41, 58). These advancements highlighted the promising role of radiomics in leveraging imaging data to enhance the evaluation and prediction of lymphatic metastasis, thereby contributing to more effective clinical decision-making and personalized treatment approaches.

CT, MRI, and ultrasound imaging had commonalities and differences in the application of radiomics for the assessment of lymphatic metastasis in clinical oncology. They all involved extracting quantitative features to evaluate tumor characteristics and lymph node involvement. These imaging modalities aimed to provide personalized medical care by offering more precise risk assessment and treatment planning.

In terms of differences, CT provided high spatial resolution and contrast, making it suitable for detecting small lymph node metastases. MRI exceeded in soft-tissue contrast and anatomical detail, making it suitable for evaluating deep-seated lymph nodes. Ultrasound, on the other hand, offered real-time imaging and radiation-free advantages, but had a relatively lower resolution. Additionally, ultrasound was cost-effective and easily accessible compared to computed tomography (CT) and magnetic resonance imaging (MRI), which were more expensive and typically used for detailed evaluations and complex cases.

Regarding to tumor types, CT was well-suited for assessing lymphatic metastasis in tumors that require high-resolution imaging, such as lung cancer, and gastrointestinal tumors. MRI was suitable for tumors that require better soft tissue contrast and anatomical details, such as breast cancer and head and neck tumors. Ultrasound was appropriate for assessing lymph node metastasis in superficial tumors such as thyroid cancer and serves as a routine screening tool.

Radiomics, a rapidly evolving field in medical imaging, held immense promise for enhancing the clinical assessment and treatment of tumor lymphatic metastasis. The integration of AI techniques with radiomics had revolutionized the analysis of medical images, offering a non-invasive and personalized approach for understanding disease progression and treatment response. As shown in Figures 5B,C, in the analysis of keyword burst intensity, artificial intelligence-related keywords such as deep learning and machine learning had emerged frequently in recent years. Artificial intelligence algorithms, particularly machine learning (ML) and deep learning (DL) models, had significantly advanced the analysis of radiomic features extracted from medical images to predict and characterize lymphatic tumor metastasis. These artificial intelligence-driven approaches enabled the identification of subtle imaging biomarkers associated with lymph node status and tumor progression, providing valuable insights for oncologists in treatment planning and monitoring (57, 58). Moreover, artificial intelligence-based radiomics models had shown promising results in predicting patient outcomes, such as survival rates and treatment responses, based on imaging data, contributing to personalized medical strategies (52, 59, 60). Furthermore, artificial intelligence tools had facilitated the automation of the whole process of radiomics, including image segmentation, feature extraction, selection, and classification processes, streamlining the analysis of large-scale imaging datasets and enhancing the efficiency of radiomic analysis (12, 61, 62). As the critical step, segmentation involved delineating regions of interest within medical images. Moreover, accurate segmentation was essential, as the quality of extracted features directly influenced the reliability of subsequent analyses and predictions. It was still controversial that if there was influence in the discriminative power of radiomic features among various segmentation methods, however, the development of ML and DL algorithms provided more possibilities for image segment, in particular several open source or commercial tools, such as PyRadiomics, and 3D Slicer, could be used to achieve the procedure (63–65). As a programming platform, 3D Slicer facilitates translation and evaluation of the new quantitative methods by allowing the biomedical researcher to focus on the implementation of the algorithm and providing abstractions for the common tasks of data communication, visualization and user interface development (66). Several years later, another architecture PyRadiomics was implemented and be used standalone or using 3D Slicer, to address the issue that lack of standardized algorithm definitions (67). Moreover, for instance, studies had demonstrated that DL models could outperform traditional segmentation methods in delineating tumors in various imaging modalities, such as U-net, context encoder network, Resnet, and attention U-net (68). Convolutional neural networks (CNNs) had emerged as a powerful tool for image segmentation tasks, and these networks could learn complex patterns and features from large datasets, enabling them to perform segmentation with high accuracy and consistency (47, 69, 70). Therefore, the integration of AI into segmentation processes had several advantages. First, it significantly reduced the time required for segmentation, allowing for faster analysis and decision-making in clinical settings. Second, AI-driven segmentation could minimize inter-observer variability, leading to more standardized and reproducible results. This is particularly important in radiomics, where the extraction of features from segmented regions was critical for building predictive models. Third, AI algorithms could be trained on large datasets, enabling them to generalize well across different populations and imaging conditions. Moving beyond that, the integration of artificial intelligence in radiomics had led to the development of predictive models that could stratify patients based on the risk of lymphatic metastasis and guide clinicians in making informed decisions regarding patient management and therapy selection (8, 40, 71).

Among the top 10 cited publications, as shown in Table 3, more citations occurred from 2021 to 2023, with 670–717 citations, which might be related to the increasing number of publications in recent years. Moreover, three articles related to DL were published in 2020 (15, 34, 35), and gained relatively more citations, suggesting that it might be more acceptable than machine learning and other algorithms. Nevertheless, with the great interest in the field of radiomics, the limitation of the reproducibility and robustness of radiomics studies appeared more obviously, due to lack of standardization in feature definition and calculation. To address this limitation, an international collaboration of 19 teams from 8 countries was initiated, named as image biomarker standardization initiative (IBSI),^1^ to establish a comprehensive radiomics workflow description, and provide benchmarking of features extraction and image process steps, as well as reporting guidelines (72). With the standardization, it would accelerate the process that translation of radiomic models into clinical practice and bring radiomics closer to clinical deployment. A free IBSI-compliant software, developed upon image biomarker explorer (IBEX), was assessed to be both easy to use and quantitatively accurate (73).

In multidisciplinary research, apart from medicine, biochemistry, genetics, and molecular biology, publications were also related to computer science and engineering, as shown in Table 3. Moreover, some studies had assessed the treatment response (9), and therapeutic implications of immune checkpoint blockade (74), which were associated with the subject area of immunology and microbiology, pharmacology, toxicology (75), and pharmaceutics. The development of this area might become more versatile in the future.

## 5 Conclusion

With the help of the R software package and VOSviewer, a global understanding of the research development, hotspots, and future trends of radiomics for evaluating lymph node status in oncology had been achieved over the past 9 years. The annual growth rate was 100.77% and the average age of the documents was 2.55. In conclusion, while CT, MRI, and ultrasound shared the objective of radiomics-based lymphatic metastasis assessment, their differences in resolution, contrast, cost, and accessibility made them more suitable for specific tumor types and clinical scenarios, and offered new opportunities for precision medicine and personalized patient care. Researchers in China and the USA contributed more than those in other countries did. The Open Access Journal *Frontiers in Oncology* occupied the top position with 160 publications. The keywords with high frequency occurrence, in the latest years, “artificial intelligence,” and “dynamic contrast enhanced computed tomography”become the emerging keywords, suggested the clinical use of novel technology and methods. Even though the interpretability of artificial intelligence models in radiomics remained a challenge, complex DL algorithms often operated as “black boxes,” making it difficult for clinicians to understand the underlying rationale behind model predictions. Enhancing the explainability and transparency of AI-driven radiomics models was essential for fostering trust among healthcare professionals and for facilitating the integration of these tools into clinical practice. Despite remarkable progress, several challenges had hindered the widespread clinical adoption of AI-driven radiomics for evaluating tumor lymphatic metastasis. One key limitation was the need for large and diverse datasets to train robust artificial intelligence models that can be generalized across different patient populations and imaging protocols. Ensuring the quality and standardization of imaging data was crucial for ensuring the reliability and reproducibility of artificial intelligence-based radiomic analyses.

## Data Availability

The raw data supporting the conclusions of this article will be made available by the authors, without undue reservation.

## Footnotes

^1^ https://arxiv.org/abs/1612.07003
